# Regulation of the Number of Cell Division Rounds by Tissue-Specific Transcription Factors and Cdk Inhibitor during Ascidian Embryogenesis

**DOI:** 10.1371/journal.pone.0090188

**Published:** 2014-03-07

**Authors:** Mami Kuwajima, Gaku Kumano, Hiroki Nishida

**Affiliations:** Department of Biological Sciences, Graduate School of Science, Osaka University, Toyonaka, Osaka, Japan; University of Minnesota Medical School, United States of America

## Abstract

Mechanisms that regulate the number of cell division rounds during embryogenesis have remained largely elusive. To investigate this issue, we used the ascidian, which develops into a tadpole larva with a small number of cells. The embryonic cells divide 11.45 times on average from fertilization to hatching. The number of cell division rounds varies depending on embryonic lineages. Notochord and muscle consist of large postmitotic cells and stop dividing early in developing embryos. Here we show that conversion of mesenchyme to muscle cell fates by inhibition of inductive FGF signaling or mis-expression of a muscle-specific key transcription factor for muscle differentiation, Tbx6, changed the number of cell divisions in accordance with the altered fate. Tbx6 likely activates a putative mechanism to halt cell division at a specific stage. However, precocious expression of Tbx6 has no effect on progression of the developmental clock itself. Zygotic expression of a *cyclin-dependent kinase inhibitor, CKI-b*, is initiated in muscle and then in notochord precursors. CKI-b is possibly downstream of tissue-specific key transcription factors of notochord and muscle. In the two distinct muscle lineages, postmitotic muscle cells are generated after 9 and 8 rounds of cell division depending on lineage, but the final cell divisions occur at a similar developmental stage. *CKI-b* gene expression starts simultaneously in both muscle lineages at the 110-cell stage, suggesting that CKI-b protein accumulation halts cell division at a similar stage. The difference in the number of cell divisions would be due to the cumulative difference in cell cycle length. These results suggest that muscle cells do not count the number of cell division rounds, and that accumulation of CKI-b protein triggered by tissue-specific key transcription factors after cell fate determination might act as a kind of timer that measures elapsed time before cell division termination.

## Introduction

The mechanisms by which embryos regulate the number of cells constituting the body are a key issue in developmental biology [1]. Control of the number of cell division rounds in specific tissues or organs is important for proper embryonic development, but its nature has remained elusive.

Eggs of the ascidian, *Halocynthia roretzi*, develop into simple tadpole larvae with a relatively small number of cells: approximately 2800. Embryonic cells divide 11.45 times on average after fertilization. Notochord and muscle cells stop dividing early during embryogenesis. Muscle in the tail comprises only 42 postmitotic cells, and 40 large postmitotic notochord cells are arrayed in a single line along the center of the tail [2]. The numbers of cell division rounds in muscle and notochord are conserved among several distantly related ascidian species, *Ciona intestinalis* and *Ascidia ahodori*, with various egg sizes [3].

At least three mechanisms regulate the number of cell division rounds during *Halocynthia* embryogenesis. This concept has been derived from a previous study in which the total numbers of cells were counted in larvae that developed from various egg fragments, in which the egg volume had been reduced by half or the egg pronucleus had been removed [4]. One of the mechanisms involves regulation by cell volume, one by the nucleocytoplasmic (N/C) ratio, and one by neither of the cell volume nor N/C ratio. When each tissue was analyzed individually, the number of cell division rounds in mesenchyme and epidermis cells appeared to be regulated by a cell volume factor. As mesenchyme cells in particular become very small after many cell divisions, it is likely that they divide until a minimum cell size limit has been reached. Cell division rounds in notochord and muscle are not affected by either cell volume or N/C ratio, implying the presence of a developmental clock. These observations suggest that the mechanisms controlling cell division are tissue-specific.

**Figure 1:**
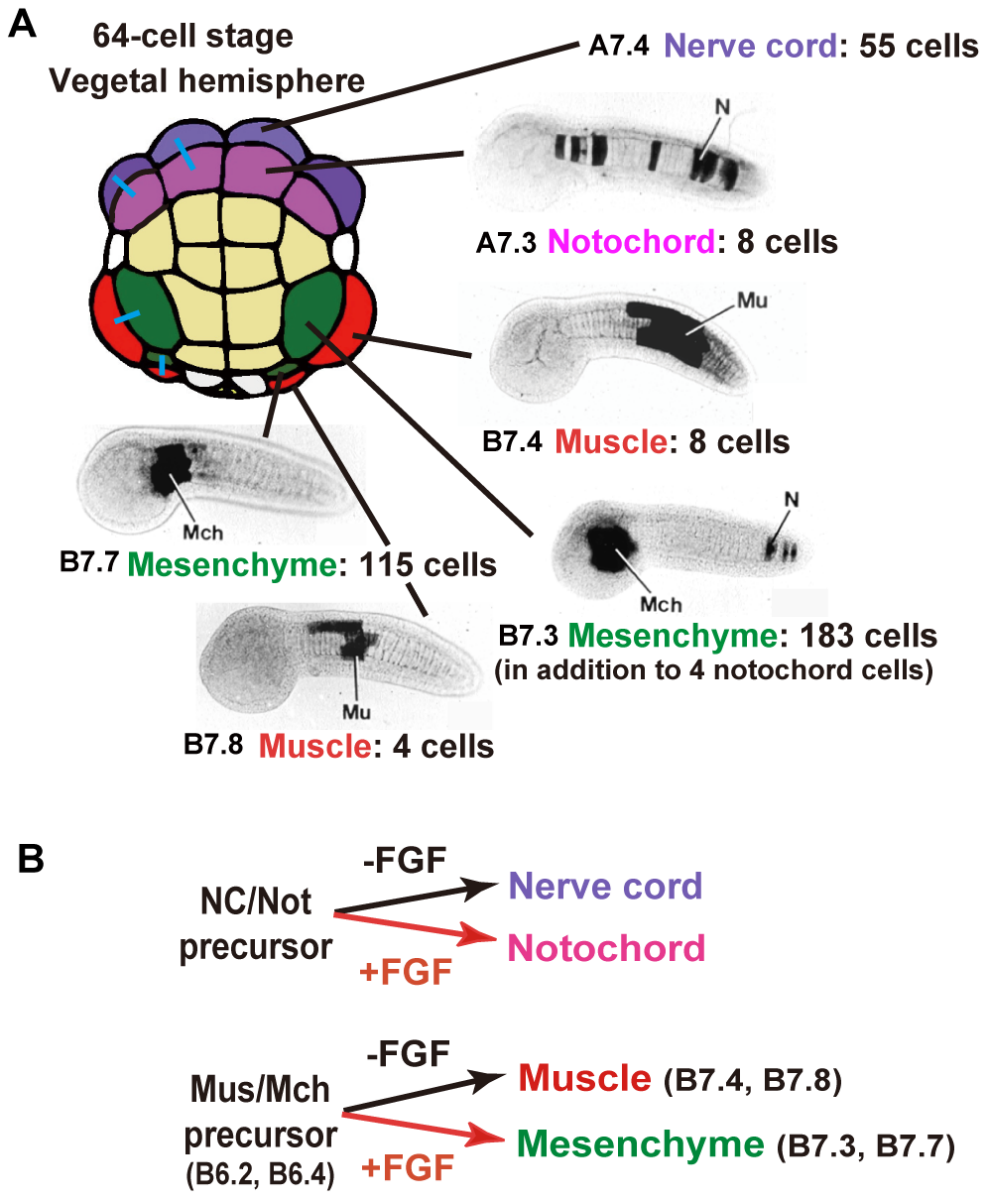
Numbers of descendant cells derived from precursor blastomeres of various tissues. (A) Vegetal view of 64-cell embryos color-coded for precursor blastomeres of each tissue. Anterior is up. Names of blastomeres and numbers of descendants cells derived from each are shown. Blue bars connect sister blastomeres on the left half of the embryo. Images showing the descendant cells are reproduced from Nishida (1987) [2]. (B) Mechanism of asymmetric cell divisions that is induced by FGF signaling in the anterior and posterior parts of embryos, respectively. Mch, mesenchyme. Mus, muscle. Not, notochord. NC, nerve cord.

A binary cell fate choice takes place between notochord and nerve cord, and between muscle and mesenchyme cells, depending on FGF signaling during the cleavage stage [5], [6] (see Fig. 1A, B). Manipulation of cell fates in notochord, nerve cord, muscle, and mesenchyme lineage cells by inhibition or ectopic activation of the inductive FGF signal results in conversion of the number of cell divisions to that of the altered fate [7]. FGF signaling in notochord promotes expression of a notochord-specific transcription factor, Brachyury (Bra), which is essential for notochord differentiation. Knockdown or mis-expression of Bra indicates that Bra is responsible for regulation of the number of cell division rounds, suggesting that Bra activates a putative mechanism to halt cell division at a specific stage. However, precocious expression of Bra does not put the developmental clock forward that controls the developmental stage at which cell division is eventually terminated [7].

Cdk inhibitors negatively regulate cell cycle progression by inhibiting the initiation of S-phase [8]–[10]. The kinase activity of Cdk/cyclin complexes is tightly regulated by binding to Cdk inhibitors. In *Drosophila* embryos, it has been reported that Dacapo, a Cdk inhibitor, is involved in the exit of embryonic cells from the cell cycle [11], [12]. Similarly, the Cdk inhibitor cki-1 facilitates transient arrest of cell division in vulval precursor cells of *C. elegans* [13], but the mechanism that determines the timing of expression or activation of Cdk inhibitors is not fully understood.

In the present study, we focused on the mechanisms that regulate the number of cell divisions in the muscle and notochord lineages. Expression of the muscle-specific key transcription factor, Tbx6 [14], which involves embryonic induction, controls the number of muscle-specific cell division rounds. It is also suggested that one of the cyclin-dependent kinase inhibitors, CKI-b, possibly located downstream of tissue-specific key transcription factors, is responsible for precocious termination of cell division in the notochord and muscle lineages.

## Materials and Methods

### Animals and embryos

Adults of the ascidian *Halocynthia roretzi* were purchased from fishermen near the Asamushi Research Center for Marine Biology, Aomori, and the Otsuchi International Coastal Research Center, Iwate, Japan. No specific permissions were required. Naturally spawned eggs were fertilized with a suspension of non-self sperm and raised in Millipore-filtered seawater containing 50 µg/ml streptomycin sulfate and 50 µg/ml kanamycin sulfate at 13°C. The embryos develop into the 64-cell stage at approximately 8 hours. Tadpole larvae hatch at 36 hours after fertilization.

Embryos were manually devitellinated with tungsten needles prior to blastomere isolation and reared in 1% agar-coated plastic dishes filled with seawater. Blastomeres were identified and isolated from embryos at the 64-cell stage with a fine glass needle under a stereomicroscope (SZX-16; Olympus). Isolated blastomeres were cultured as partial embryos and fixed with cold 70% ethanol at the hatching stage. For cell counts of the resulting partial embryos, nuclei were stained with DAPI. They were gently squashed on glass slides by compressing them with cover slips until the constituent cells spread into a monolayer [4].

To inhibit the FGF signaling pathway, embryos were treated with 2 µM U0126 (Promega) after the 16-cell stage [7], [15]. U0126 is MEK inhibitor that inhibits both the activation of MEK by Raf and the activation of ERK by MEK [16]. As controls, embryos were treated with DMSO in seawater. To inhibit cell division, cleavage was permanently arrested with 2.5 µg/ml cytochalasin B (Sigma) at the 110-cell stage [15].

### 5′ RACE of *Hr-CKI-b*

*Halocynthia roretzi* homologs of Cdk inhibitor were identified in the MAGEST database (EST database of eggs and embryos; http://magest.hgc.jp/) [17]. One of them, *Hr-CKI-b* (MAGEST clone FE003A24), has a typical Cdk inhibitor domain, and showed tissue-specific expression. Since the MAGEST clone contained only the full-length ORF and 3′ UTR sequences of *Hr-CKI-b*, its 5′ UTR sequence was obtained by 5′ RACE (GenBank Accession No. AB850878). 5′ RACE was carried out using embryonic cDNA and a BD SMART™RACE cDNA Amplification Kit (BD Bioscience Clontech), and the amplified fragment was sequenced.

Whole-mount *in situ* hybridization was performed basically as described [18]. Specimens were hybridized with the digoxigenin (DIG)-labeled *Hr-CKI-b* probe.

### Morpholino antisense oligos and mRNA

To suppress Hr-Brachyury (Bra) function, we injected about 1000 pg of a 25-mer Bra morpholino oligonucleotide (MO; Gene Tools), covering the first methionine, into fertilized eggs. The nucleotide sequence was 5′-TTGTAATTGACATAATTCCTTGTAC-3′ [7], [19]. As a control, a standard MO (Gene Tools) was injected.

*Hr-Tbx6* [14] and *Hr-CKI-b* ORFs were cloned into the RN3EX vector (RN3 vector [20] with an extra *Xho*I site). mRNAs flanked by *Xenopus* globin UTRs were synthesized from each of the plasmids using the mMessage mMachine kit and poly (A) tailing kit (Ambion). 0.1 µg/µl of *Hr-Tbx6* and 0.3 µg/µl of *Hr-CKI-b* mRNA solutions were injected into fertilized eggs. *mCherry:Tbx6* mRNA was synthesized from the RN3EX vector with an insert in which the mCherry coding sequence was inserted in-frame into the 5′ end of the *Tbx6* ORF. The synthesized mRNA had the original *Tbx6* UTRs; 0.1 µg/µl of the mRNA solutions was injected. To visualize the descendant cells of the mesenchyme precursors, mRNA encoding histone H2B protein (from the appendicularian *Oikopleura dioica*) fused with mCherry fluorescent protein was synthesized from the plasmid pSD64TF carrying the ORF (kindly provided by Dr. A. Nishino, Hirosaki University).

### Time-lapse video recording

Blastomeres isolated at the 64-cell stage were cultured in agar-coated dishes, and successive cell divisions were recorded with a BX61 motorized microscope (Olympus) equipped with a water-immersion lens and digital camera. The DIC images were acquired using LuminaVision software (Mitani Co., Tokyo, Japan) at one frame every 3 minutes for approximately 11 hours.

## Results

### Numbers of cell division rounds in various tissues

Ascidian embryos provide a good system for studying the regulation of cell division rounds during embryogenesis. The cell fate of each blastomere in 64-cell-stage embryos has been investigated in both tailbud embryos and hatched larvae [2], [7]. The number of cell division rounds varies depending upon tissue type (Fig. 1A). It has been shown that a single nerve cord (namely, posterior neural tube) precursor blastomere of the 64-cell embryo (after 6 rounds of cleavage) gives rise to 55 cells in a hatched larva, dividing 5.8 times on average after the 64-cell stage. The primary notochord precursor blastomere (A7.3 and A7.7 cells) divides three times, invariably giving rise to 8 postmitotic notochord cells. The entire tail muscle consists of 21 cells on each side, which are also postmitotic at the tailbud stage. The B7.4 cell of the 64-cell-stage embryo divides three times to give rise to the 8 muscle cells in the middle part of the tail. The B7.8 cell is smaller than the B7.4 cell because of unequal-sized cell divisions during the early cleavages [21]. It also develops into tail muscle in the anterior part, but the number of muscle cells is 4 after 2 cell divisions. Thus, all the resulting muscle cells are similar in size. It is of interest that the numbers of cell division rounds in these two muscle lineages are distinct. The other 9 muscle cells are derived from B7.5, A8.16, b8.17, and b8.19, although the cell fate of these cells are not restricted to give rise to muscle at the 64-cell stage. Mesenchyme cells in the larva are derived from the B7.3 and B7.7 blastomeres. The B7.7 cell is smaller than the B7.3 cell. The single B7.3 cell gives rise to 183 mesenchyme cells, dividing 7.5 times after the 64-cell stage, in addition to 4 secondary notochord cells at the tip of the tail [7].

It has not been clarified how many mesenchyme cells the B7.7 cell produces. To count the number of descendant cells, mRNA encoding histone H2B protein fused to mCherry fluorescent protein was injected into blastomeres, and the number of nuclei with red fluorescence was counted at the hatched larva stage using the methods described in our previous paper [7]. We found that the mesenchyme precursor gives rise to 115±10.7 (mean±s.e.m., n = 3) mesenchyme cells, corresponding to 6.8±0.13 ( = log_2_ 115) cell divisions on average (n = 3, Fig. 1A and Fig. S1). These mesenchyme cells continue to divide during metamorphosis and contribute to the adult body [22].

### Changes in the number of cell division rounds according to cell fate change

Our previous studies have demonstrated that isolated tissue precursor cells undergo nearly comparable number of cell divisions relative to their counterparts in normal whole embryos [4], [7], [23]. We utilized this system in the following experiments to investigate the regulation of cell division. Counting of cell descendants from a specific blastomere is easier in partial embryos than in whole larvae. This approach makes it possible to exclude any external influences on cell division, as the partial embryos develop in isolation.

We have been interested in the difference in the number of cell divisions between two muscle lineages, B7.4 and B7.8, that give rise to 8 and 4 postmitotic muscle cells, respectively. In the posterior region of embryos, mesenchyme/muscle mother cells (B6.2) of 32-cell embryos divide asymmetrically into mesenchyme (B7.3) and muscle (B7.4) progenitor cells, while the other mesenchyme/muscle mother cells (B6.4) of 32-cell embryos similarly divide into mesenchyme (B7.7) and muscle (B7.8) progenitor cells (Fig. 1B). These asymmetric cell divisions are mediated by FGF induction [24].

**Figure 2:**
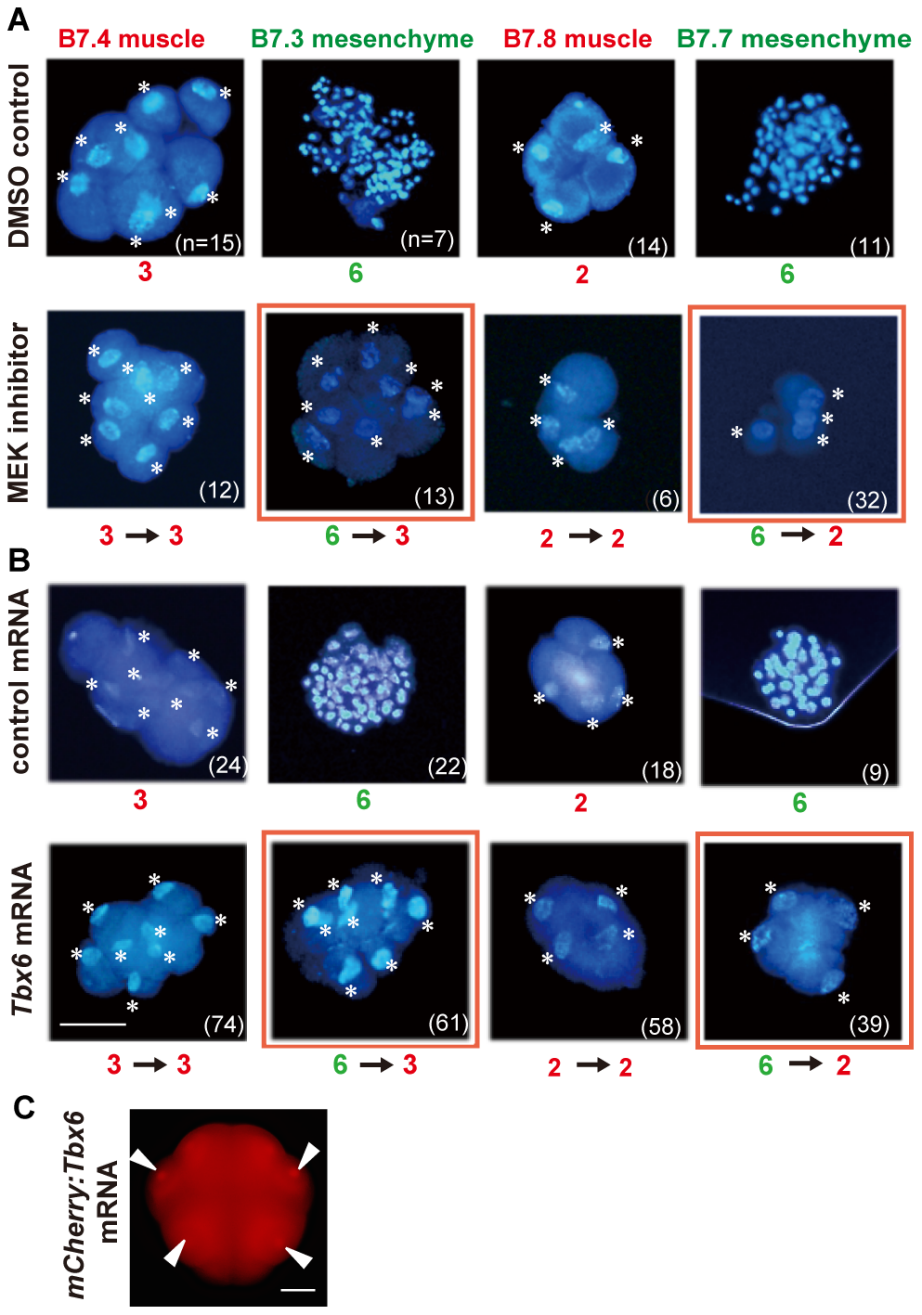
Inhibition of FGF signaling by MEK inhibitor. (A) Muscle and mesenchyme blastomeres were isolated from 64-cell embryos treated with DMSO as a control (upper row), and with MEK inhibitor (bottom). Resulting partial embryos were gently squashed on glass slides by compressing them with cover slips until the constituent cells spread into a monolayer. Nuclei (asterisks) were stained with DAPI, and numbers of constituent cells were counted. The number of cell divisions was then calculated. Approximate numbers of cell division rounds in controls and their changes resulting from treatment are indicated below the photos. Numbers of partial embryos observed are shown in parentheses. Specimens that showed altered numbers of cell divisions are indicated by red squares. For detailed data see Table 1. (B) Muscle and mesenchyme blastomeres were isolated from 64-cell embryos that were injected with *H2B:mCherry* mRNA as a control (upper row), and with *Tbx6* mRNA (bottom). For detailed data see Fig. 3. (C) Synthetic mRNA encoding the mCherry:Tbx6 fusion protein with the original UTRs of *Tbx6* mRNA was injected into fertilized eggs. Nuclear fluorescence was detected as early as the 16-cell stage. Scale bar, 50 µm.

It is possible to convert cell fates through manipulation of inductive cell interaction by FGF (Fig. 1B). Mesenchyme development requires induction by FGF at the 32- to early 64-cell stage. FGF acts as an induction factor that determines cell fate, and not as a growth factor [24], [25]. Inhibition of FGF signaling has been achieved by treatment with U0126, an inhibitor of MEK, a member of the Ras/Raf/MEK/MAPK cascade [16]. Treatment with U0126 is sufficient to convert mesenchyme precursors to muscle precursors [15]. Embryos were treated from the 16-cell stage onward, and tissue precursor blastomeres were isolated at the 64-cell stage. The numbers of cells in the resulting partial embryos were counted at the hatching stage by staining of nuclei with DAPI. A previous study [7] has shown that when B.7.3 mesenchyme precursors are converted to muscle, they only divide 3 times, which is comparable to the B7.4 muscle cell. First, we confirmed and reproduced this result (Fig. 2A and Table 1; B7.3 mesenchyme). Treatment with the control solvent, DMSO, did not affect the number of cell division rounds. The B7.7 mesenchyme precursor was then treated with U0126, and it was found to divide only twice (1.9±0.04 s.e.m.), being comparable to the B7.8 muscle cell (Fig. 2A and Table 1; B7.7 mesenchyme). Without the treatment, isolated mesenchyme precursors divided approximately 6 times in isolation, although this is slightly less than the number of cell divisions in whole intact embryos (6.4 and 6.2 times in isolation compared to 7.5 and 6.8 times in whole embryos for B7.3 and B7.7, respectively (Figure 1A)). Cell division rounds of muscle precursors, whose cell fate does not depend on FGF signaling, were not altered by U0126. Thus, cell fate conversion resulted in a change in the number of cell divisions according to the altered fate.

**Table 1:** Cell counts of partial embryos treated with MEK inhibitor.

Blastomeres	MEK inhibitor (fate)	n	Cell count	Cell divisions
B7.4 Muscle	+ (Mus)	12	7.3±0.2	2.9±0.04
	− (Mus)	15	7.4±0.3	2.9±0.07
B7.3 Mesenchyme	***+ (Mus)***	13	7.3±0.3	***2.8±0.08***
	− (Mch)	7	87.4±7.3	6.4±0.1
B7.8 Muscle	+ (Mus)	6	3.8±0.2	1.9±0.07
	− (Mus)	14	3.7±0.1	1.9±0.05
B7.7 Mesenchyme	***+ (Mus)***	32	3.7±0.1	***1.9±0.04***
	− (Mch)	11	78.1±6.2	6.2±0.1

DMSO, the solvent of MEK inhibitor, was used as a control.

Cell counts and numbers of cell divisions are presented as mean ± s.e.m.

Conditions are highlighted in bold and italic letters when cell fate changes are expected.

Numbers of cell divisions are highlighted in bold and italic letters when a change in the number was observed.

Mch, mesenchyme. Mu, muscle.

These results indicate that when mesenchyme fate is converted to muscle fate, the number of cell division rounds becomes similar to that of each counterpart sister muscle cell. Therefore, even if the cell fate is muscle in both lineages, the regulation of cell divisions is distinct, and dependent on lineage. It is likely that lineage-specific differences are already present in the mother cells of 32-cell embryos, B6.2 and B6.4 cells.

### Tbx6 controls both cell differentiation and cell division rounds

After mesenchyme fates have been determined by an extracellular inductive signal, it is not possible to exogenously manipulate cell fates any further. However, it is still possible to interfere with cell differentiation processes by manipulation of transcription factors within the cells. The key transcription factor for muscle differentiation in ascidians is Tbx6, and its presence is sufficient to promote muscle differentiation to some extent when expressed ectopically [14], [26], [27]. In *Halocynthia*, weak expression of the *Tbx6* gene is first detected in a pair of endoderm precursor blastomeres at the 32-cell stage, although the significance of this endodermal expression is not known. Then, exclusive expression starts in muscle lineage blastomeres at the 64-cell stage [28], downstream of the maternally supplied muscle determinant, macho-1 [29]. We next examined whether mis-expression of Tbx6 would interfere with the number of cell divisions in mesenchyme cells.

**Figure 3:**
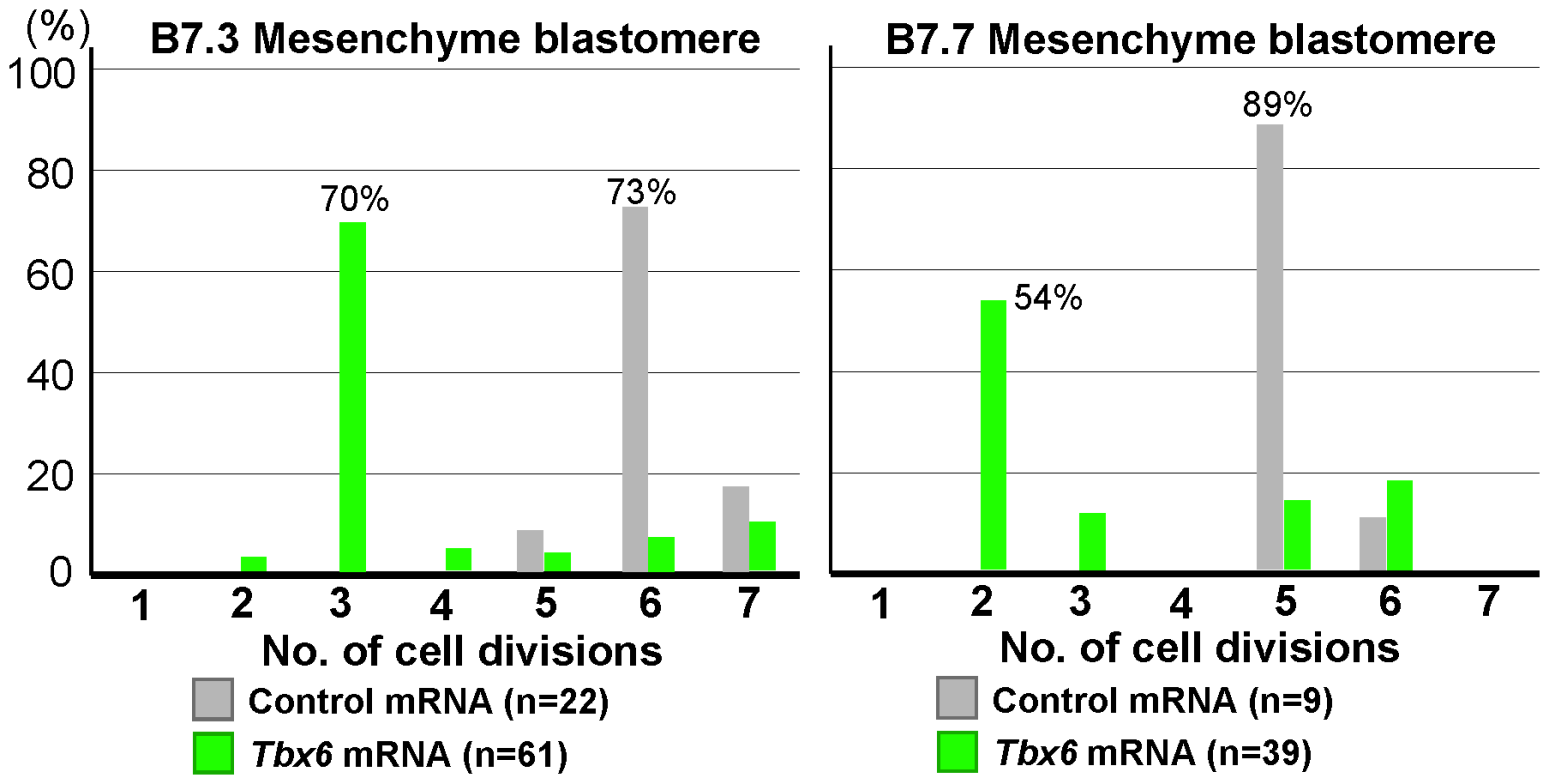
Numbers of cell divisions of mesenchyme cells in Tbx6-mis-expressing embryos. B7.3 and B7.7 mesenchyme blastomeres were isolated from 64-cell embryos that had been injected with control *H2B:mCherry* mRNA (gray bars) and *Tbx6* mRNA (green bars). The numbers of descendant cells were counted, and the numbers of cell divisions were then calculated. On the abscissa, e.g., 3 cell divisions represents partial embryos that divided 2.50 to 3.49 times. Numbers of partial embryos observed are indicated in Fig. 2B. Proportions of major specimens are given above the columns.

Fertilized eggs were injected with *Tbx6* mRNA. The impact of mis-expression of Tbx6 on the number of cell divisions in mesenchyme cells was similar to that of treatment with the MEK inhibitor (Fig. 2B and 3). The majority of isolated B7.3 mesenchyme blastomeres from 64-cell embryos subjected to mis-expression divided three times. Those of B7.7 blastomeres divided twice. Injection of control *H2B-mCherry* mRNA had no effect. The number of cell divisions resulted from the mis-expression was variable (Fig. 3). Injected mRNAs diffuse slowly, and are occasionally inherited by only one daughter blastomere at the first cleavage [30]. This could be one explanation for the observed variation. These results suggest that the key muscle transcription factor, Tbx6, controls the number of cell divisions as well as cell differentiation, as has been shown in ascidian notochord where the transcription factor Brachyury also controls both cellular cascades [7]. Tbx6 likely activates a mechanism that regulates muscle-specific cell division rounds.

On the other hand, when mesenchyme fate is converted to muscle fate by Tbx6, the numbers of cell divisions of B7.3 and B7.7 mesenchyme become close to that of each counterpart sister muscle cell, B7.4 and B7.8, respectively. Again, this suggests that there is lineage-specific control of cell divisions, which is independent of the muscle differentiation cascade and not simply downstream of Tbx6.

The stage of initiation of *Tbx6* expression did not appear to determine when cell division ceases because injection of *Tbx6* mRNA at the 1-cell stage did not affect the number of cell divisions in the original muscle precursors (Fig. 2B and 3). In normal embryos, muscle-specific *Tbx6* gene expression starts at the 64-cell stage [28]. Fertilized eggs injected with *Tbx6* mRNA never ceased cell division during the cleavage stages, and then muscle precursors divided 2 or 3 times after the 64-cell stage, as in normal embryos. Precocious initiation of *Tbx6* expression does not influence the number of total cell division rounds in muscle cells. Therefore, precocious *Tbx6* expression from the 1-cell stage does not put the developmental clock forward, but makes cells exit from the cell cycle at an appropriate time, probably defined by the Tbx6-independent clock or timekeeper mechanism. Translation or nuclear transportation of Tbx6 protein might be temporally regulated and unable to start before a certain stage. To test this possibility, synthetic mRNA encoding the mCherry:Tbx6 fusion protein with the original UTRs of *Tbx6* mRNA was injected into fertilized eggs. Red fluorescence within nuclei was first detected at the 16-cell stage (20 out of 22 cases; Fig. 2C), indicating that Tbx6 protein was translated from the injected mRNA and then transported into the nucleus before the initiation of endogenous *Tbx6* gene expression at the 64-cell stage in muscle precursor blastomeres. Thus, it seems unlikely that there is specific temporal regulation of translation or nuclear transportation of Tbx6 protein. This situation is similar to that found in a previous study of notochord formation, which is regulated by the transcription factor Brachyury [7].

### Cell cycle lengths differ in B7.4- and B7.8-lineage muscle cells

**Figure 4:**
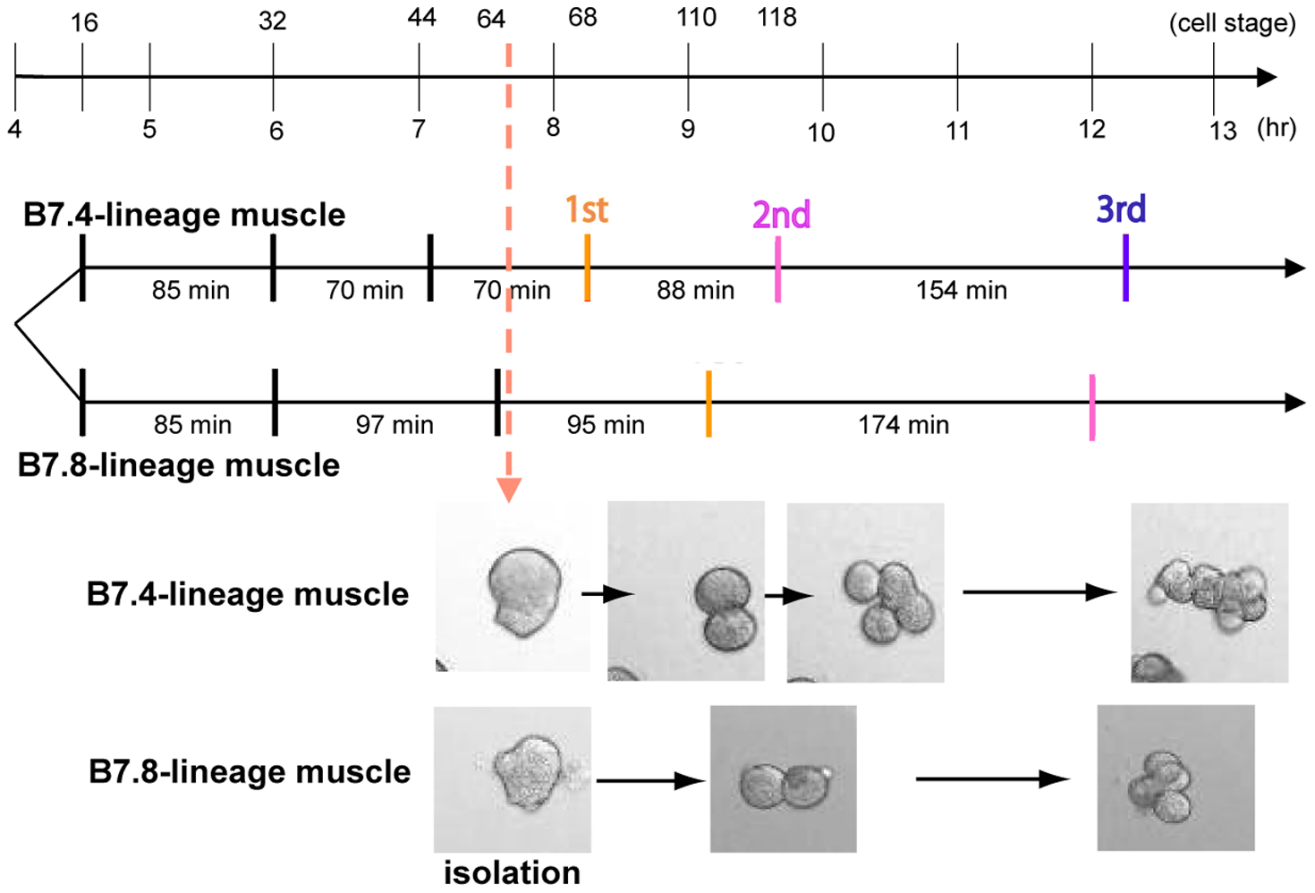
Timing of cell divisions in the B7.4 and B7.8 muscle lineages. Progression of cell division was monitored by time-lapse video. The B7.4 and B7.8 muscle precursors were isolated from 64-cell embryos and recorded. Snapshots from the resulting time-lapse video are shown at the bottom. The duration of each cell cycle up to the 64-cell stage was observed in whole embryos. The duration of each cell cycle after the 64-cell stage was averaged from 5 independent time-lapse recordings. See also Movie S1.

To gain insight into the differences between the B7.4- and B7.8-lineage muscle cells, cell division sequences were monitored using time-lapse movies. At the 64-cell stage, B7.4 and B7.8 cells were isolated and recorded up to the terminal cell division together with whole embryos (Fig. 4 and Movie S1). The B7.4 and B7.8 lineages are separated at the 16-cell stage. The cell cycle of the B7.8 lineage became longer than that of the B7.4 lineage after the 32-cell stage. In both lineages, the final cell divisions before reaching a postmitotic state were observed around 12 hr after fertilization. Therefore, both lineages terminate cell division at a similar developmental time point, although B7.4 divides three times, while B7.8 divides twice after the 64-cell stage. The difference in the number of cell divisions seems to be achieved by differences in cell cycle length rather than the stage of terminal cell division. Therefore, we assumed that a certain common process is running to terminate cell division in both the B7.4 and B7.8 muscle lineages at a similar developmental stage.

### Expression of *Cdk* inhibitor starts in notochord and muscle lineage cells

Cdk inhibitors negatively regulate cell cycle progression by inhibiting the initiation of S-phase [8]–[10]. Therefore, Cdk inhibitor expression could be a candidate for such a common process. In the genome of another ascidian species, *Ciona intestinalis*, two Cdk inhibitor genes are present [31]. Using the sequences of these *Ciona* genes, we sought *Halocynthia roretzi* Cdk inhibitor homologs in the MAGEST database (EST database of eggs and embryos; http://magest.hgc.jp/) [17]. There were two candidates corresponding to *Ci-CDKI-a* and *Ci-CDKI-b* of *Ciona*, respectively, with bidirectional best hits. Domain analysis showed that both *Hr-CKI-a* (MAGEST clone FE004D21) and *Hr-CKI-b* (MAGEST clone FE003A24) have a typical Cdk inhibitor domain. *Hr-CKI-a* did not give a clear *in situ* hybridization signal during embryogenesis, whereas *Hr-CKI-b* was expressed in notochord and muscle. We obtained the complete cDNA sequence of *Hr-CKI-b* by resequencing the MAGEST clone, and by independent 5′ RACE (GenBank Accession No. AB850878).

**Figure 5:**
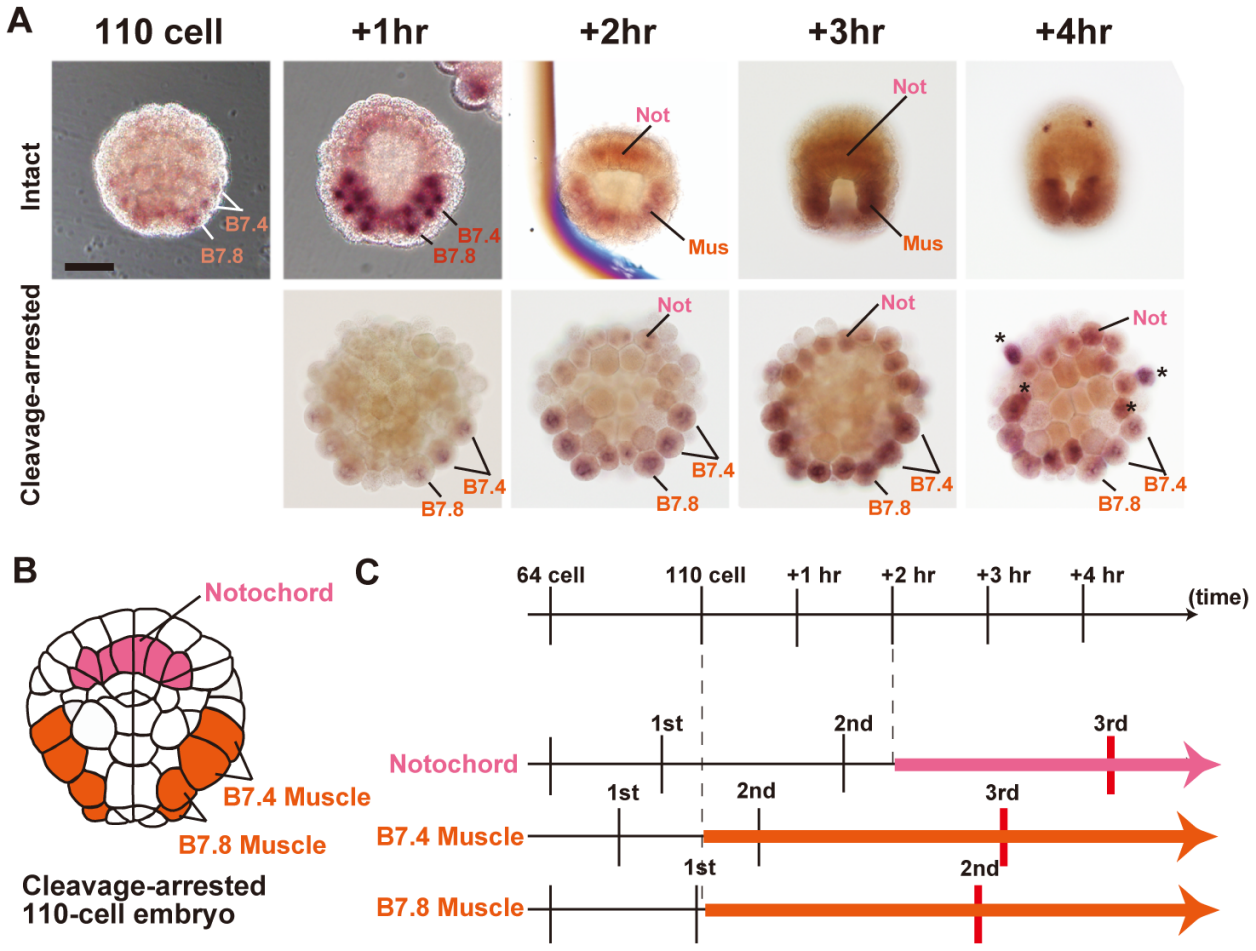
*CKI-b* gene expression. (A) (upper row) *In situ* hybridization with the *Hr-CKI-b* probe. The expression becomes evident at the 110-cell stage in the B7.4 and B7.8 muscle lineage precursors simultaneously. At the 110-cell stage plus one hour, there are 4 B7.4 descendants and 2 B7.8 descendants. At 2 hours after the 110-cell stage, the expression has become evident in notochord precursors. The stages from the 110-cell stage through the gastrula and up to the neural plate stage are shown. (bottom) Expression of *CKI-b* in embryos whose cleavages were arrested at the 110-cell stage. Asterisks indicate *de novo* expression at the 110-cell stage plus 4 hours, which probably corresponds to the heavily stained cells in the above photo. (B) Schematic representation of the arrested 110-cell embryos, showing the position of each tissue precursor cell. Vegetal view. (C) Timetable of *CKI-b* expression and cell division progression after the 64-cell stage. Mus, muscle. Not, notochord.

*CKI-b* expression was first detected at the 110-cell stage in muscle precursors of the B7.4 and B7.8 lineages at the same time (Fig. 5A). The expression was maintained up to the neural plate stage. Expression was also observed in notochord precursors starting from 2 hours after the 110-cell stage. To precisely identify cells of the *CKI-b*-expressing lineage, cell division was arrested at the 110-cell stage using an actin depolymerization agent (Fig. 5A and B). Expression in the muscle and notochord cells was thus confirmed. Larval notochord and muscle cells are large, and stop dividing early during embryogenesis. After fertilization, notochord cells divide only 9 times, B7.4-lineage muscle cells divide 9 times, and B7.8-lineage muscle cells divide 8 times. Therefore, expression of the Cdk inhibitor in muscle and notochord is consistent with the early termination of cell division. The timetable of *CKI-b* expression and cell division progression after the 64-cell stage is summarized in Fig. 5C. Expression in notochord lags behind that in muscle by 2 hours, and this seems well consistent with the delayed final cell division in notochord. Interestingly, cell divisions still continue once or twice after the initiation of *CKI-b* gene expression.

**Figure 6:**
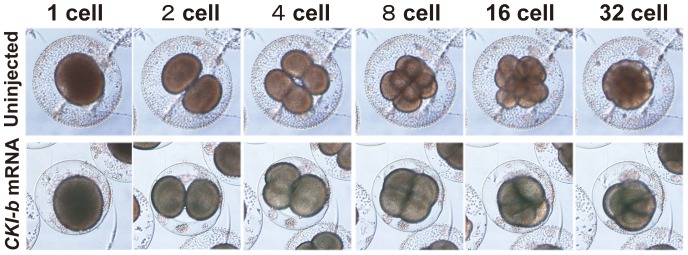
Overexpression of CKI-b inhibits cleavage. *CKI-b* mRNA was injected into eggs at approximately one hour after fertilization (bottom row). The cell cycle was prolonged at the 4-cell stage relative to normal embryos (upper), and most of them ceased dividing at the 8- or 16-cell stage.

To confirm the activity of CKI-b, synthetic mRNA was injected into eggs at approximately one hour after fertilization. The cell cycle was prolonged at the 4-cell stage, and most of the cells ceased dividing at the 8- or 16-cell stage (Fig. 6). It seems to take approximately 2 hours for accumulation of a sufficient amount of CKI-b protein translated from the injected mRNA. This could explain the time lag between the initiation of the CKI-b gene expression and the final cell division observed in normal embryos (Fig. 5C), although the amount of the injected mRNA will be different from that of the endogenous CKI mRNA and the activity of the translational machinery might be different between earlier and later stages. We do not know when the cells enter the final S-phase, but such a 2-hour time lag would well explain the discrepancy.

### Expression of *CKI-b* occurs downstream of inductive cell interaction and the key transcription factor

**Figure 7:**
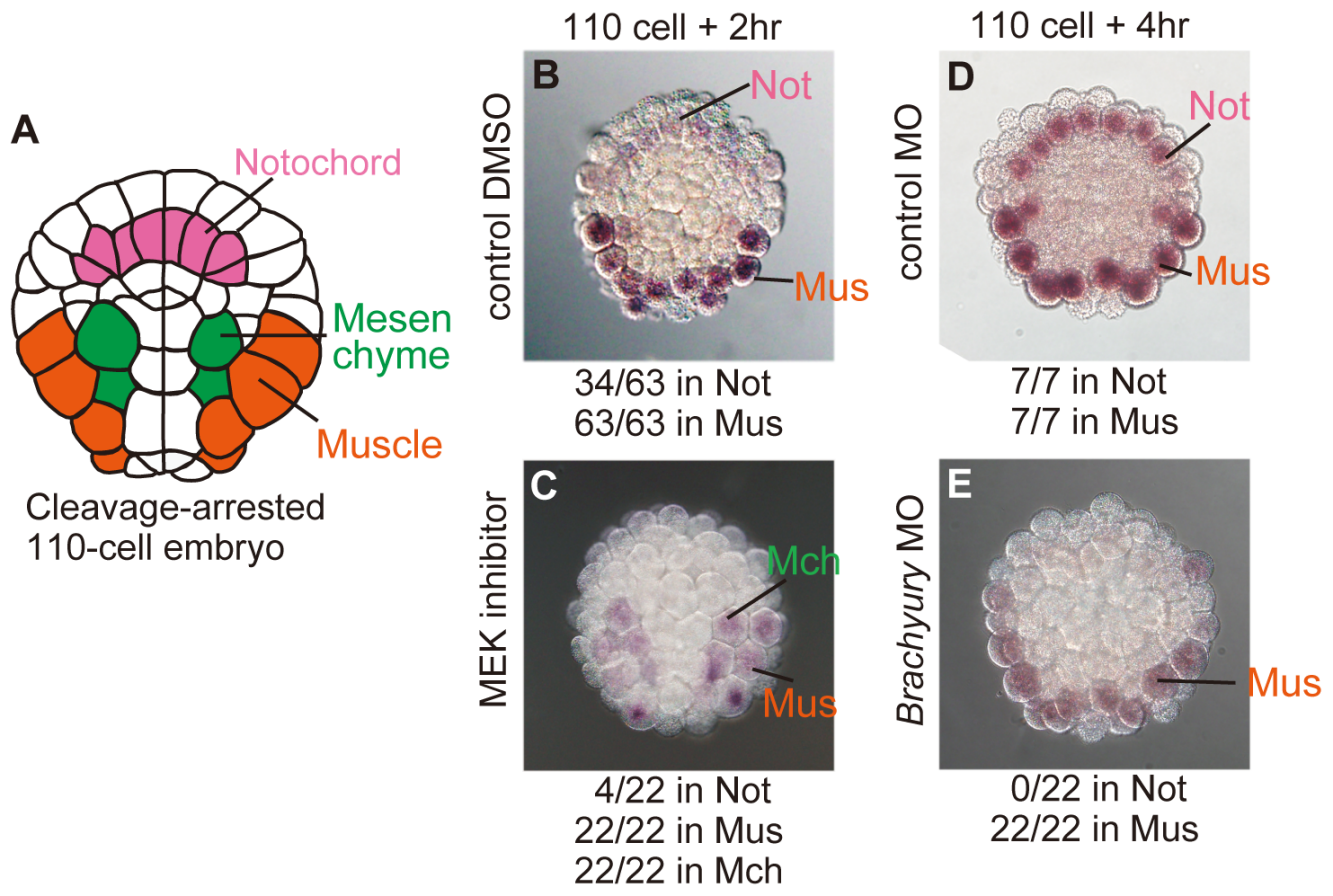
Expression of *CKI-b* occurs downstream of inductive cell interaction and the key transcription factor. (A) Schematic representation of arrested 110-cell embryos, showing the position of each tissue precursor cell. Vegetal view. (B) *CKI-b* expression in a cleavage-arrested 110-cell embryo treated with DMSO as a control. (C) That treated with MEK inhibitor. Expression in the notochord was lost, and ectopic expression was evident in mesenchyme cells. (D) *CKI-b* expression in a cleavage-arrested 110-cell embryo injected with control MO. (E) That in an embryo injected with Brachyury MO. The expression in notochord cells was lost. Numbers of embryos that showed the expression in each tissue per those of observed embryos are shown. Mch, mesenchyme. Mus, muscle. Not, notochord.

To investigate the regulation of *CKI-b* expression, embryos were first treated with MEK inhibitor to suppress embryonic induction by FGF signaling. In these embryos, notochord transfates to nerve cord and mesenchyme is converted to muscle (Fig. 1B). Accordingly, *CKI-b* expression in notochord was lost, and expressed ectopically in mesenchyme (Fig. 7A, B, and C), indicating that *CKI-b* expression is regulated by cell fate specification mechanisms involving inductive FGF signaling.

We then tested whether *CKI-b* expression is regulated by key transcription factors. For this, we used a Brachyury (Bra) morpholino antisense oligo (MO), the specificity of which had been confirmed by a rescue experiment [7]. Bra is the key transcription factor for notochord development, and its expression is activated by FGF signaling (Fig. 1B). It controls notochord differentiation and cell division rounds. Unfortunately, Tbx6 MO did not work well as muscle differentiation was only partially suppressed by the Tbx6 MO. This is because the MO does not efficiently suppress translation. There is another possibility for the reason. *Ciona* has at least three paralogous genes, *Tbx6 a/b/c* [27]. Similarly, *Halocynthia* could have several Tbx6 paralogues. Without Bra, notochord cells continue cell division after normal terminal cell division [7]. Knockdown with the Bra MO resulted in abrogation of *CKI-b* expression in notochord cells but not in muscle cells, showing that *CKI-b* expression lies downstream of the key transcription factor in cells of notochord lineage.

## Discussion

The numbers of cell division rounds during embryonic development vary according to cell lineage. We focused on the mechanisms that regulate the number of cell divisions in the two muscle lineages and notochord lineage during ascidian embryogenesis. Notochord and muscle cells are large, and stop dividing early during embryogenesis. Notochord cells divide only 9 times, B7.4-lineage muscle cells divide 9 times, and B7.8-lineage muscle cells divide 8 times after fertilization, producing differentiated postmitotic cells.

### Numbers of cell divisions depend on inductive cell interaction and a key transcription factor

When cell fates were converted by manipulation of inductive FGF signaling, the number of cell division rounds was also changed in accordance with the altered cell fate. This suggests that future cell division rounds are flexible, and not determined before the stage of inductive events. We also showed that, in the absence of induction, the intrinsic key transcription factor in muscle, Tbx6, is involved in both cell differentiation processes and control of the number of cell cycle rounds. This accords well with previous analysis of the regulation of cell division number during notochord formation, which is regulated through the presence of induction by FGF and the key transcription factor, Bra [7].

Injection of *Tbx6* mRNA into eggs, i.e. precocious expression of *Tbx6*, did not affect the number of cell divisions in original muscle precursors. Therefore, the stage of initiation of *Tbx6* expression did not appear to determine when cell division ceases. The result of injection of mRNA encoding the Tbx6:mCherry fusion protein indicated that Tbx6 protein was translated from the injected mRNA and transported into the nucleus, well before the appearance of expression of the endogenous *Tbx6* gene. Tbx6 would directly or indirectly activate a mechanism, whose timing of action is regulated by a Tbx6-independent developmental timekeeper, in order to halt cell division at the correct time point. Tbx6 does not govern the progression of the developmental timekeeper. A similar result has been obtained using precocious expression of Bra. Injection of *Bra* mRNA at the one-, 4-, and 16-cell stages did not affect the number of cell divisions in original notochord precursors [7]. At the moment, the results of this and previous studies tell us almost nothing certain about the molecular mechanism of the timekeeping mechanism.

### Differences in two distinct muscle lineages

The larger B7.4 muscle precursor cells divide 3 times and the smaller B7.8 muscle precursors divide twice after the 64-cell stage. Consequently, all the resulting muscle cells in the larval tail are similar in size. One possibility is that muscle cells can sense their individual volume and cease dividing when this volume falls below the threshold, but this seems unlikely. When unfertilized eggs are bisected into halves, they develop with a normal cleavage pattern and develop into morphologically normal-looking dwarf larvae. The half-sized dwarf larvae have normal numbers of tail muscle and notochord cells with a normal spatial arrangement [4]. It is likely that the volume of the muscle cells is simply half, because embryos do not feed during embryogenesis. On the other hand, when two-cell embryos are dissociated into two blastomeres, they develop into left- or right-half larvae with half the number of notochord cells, although the number of muscle cells in these half larvae has not been reported.

When mesenchyme fate is converted to muscle fate by suppression of FGF signaling and mis-expression of Tbx6, the number of cell divisions approximates that of each counterpart sister muscle cell. Thus, lineage-specific control of cell divisions appears to operate. Lineage-specific differences are likely to be already present in the muscle/mesenchyme mother cells of 32-cell embryos, B6.2 and B6.4 cells. The lineage-specific difference is independent of the muscle differentiation cascade, and is not simply downstream of Tbx6. The terminal cell divisions in both lineages occurred at a similar developmental time point. It is likely that a common process is simultaneously running to terminate cell division in both muscle lineages at a similar developmental stage. The difference in the number of cell divisions seems to be attributable to the cumulative difference in cell cycle length, rather than by the difference in the stage of terminal cell divisions.

It is unclear what is responsible for the difference in cell cycle length between the two muscle lineages. In ascidians, a dozen posteriorly localized maternal mRNAs known as *postplasmic/PEM* mRNAs have been identified [32]–[34]. These RNAs are known to control unequal cell divisions, cell fates, and germ cell development in the posterior region of ascidian embryos. B7.8 is positioned posteriorly and B7.4 is located more laterally. It is possible that some of the postplasmic/PEM mRNAs are involved in creating the difference in cell cycle length between the B7.4 and B7.8 lineages.

### Possible role of CKI-b in precocious termination of cell division in notochord and muscle cells

*CKI-b* expression was initiated at the 110-cell stage, but was delayed by 2 hours in notochord relative to that in muscle precursors. This difference seems to correspond well with the difference in timing of the final cell divisions in muscle and notochord. However, cells still divide once or twice even after the initiation of *CKI-b* gene expression. One possibility is that CKI activity or stability might be temporally regulated through post-translational modification [35], [36]. When we injected *CKI-b* mRNA into eggs, it took approximately 2 hours to impede and eventually inhibit cell division. Thus another possibility is that a certain period needs to elapse in order for a sufficient amount of CKI-b protein to accumulate via translation from mRNA. This could explain the time lag between the initiation of gene expression and the final cell division observed in normal embryos.

*CKI-b* expression was initiated simultaneously at the 110-cell stage in muscle precursors of the B7.4 and B7.8 lineages. If accumulation of CKI-b protein shows the same temporal progression in both lineages, this could potentially be the common process operating to terminate cell division at a similar stage, supporting the idea that the difference in the number of cell divisions is due to the cumulative difference in cell cycle length.

Expression of *CKI-b* occurs downstream of inductive cell interaction and a key transcription factor. The presence and absence of FGF signaling would indirectly affect *CKI-b* expression via activation of the expression of key transcription factors [37] because the presence of FGF signaling promotes *CKI-b* expression in notochord cells, whereas its absence promotes *CKI-b* expression in muscle cells in normal embryos (see Fig. 1B and 5). We showed that Bra is pivotal for *CKI-b* expression. It is uncertain whether Tbx6 is also involved in *CKI-b* expression, but it seems likely, as both Bra and Tbx6 are T-box transcription factors [38]. There is an intriguing possibility that *CKI-b* expression is upregulated using common T-box-binding cis-regulatory elements in notochord and muscle. Thus, similar mechanisms appear to operate in both notochord and muscle. Expression of the tissue-specific key transcription factors, Bra and Tbx6, regulated by the presence and absence of embryonic induction that determine cell fates, is involved in regulation of the number of cell division rounds specific to notochord and muscle. CKI-b, whose expression is triggered by tissue-specific key transcription factors, could be responsible for the early termination of cell division in notochord and muscle cells. These results suggest that muscle and notochord cells do not count the number of cell division rounds, and that accumulation process of CKI-b protein after cell fate determination would act as a kind of timer that measures elapsed time from initiation of *CKI-b* expression to termination of cell division.

## Supporting Information

Figure S1 **Number of descendant cells of the B7.7 mesenchyme precursor.** B5.2 blastomere of the 16-cell embryo was labeled with H2B:mCherry. It gives rise to B7.7 mesenchyme (square bracket), B7.5 and B7.8 muscle cells (6 arrowheads), and B7.6 primordial germ cells (two arrows). The larva was squashed to count the number of nuclei. Mesenchyme cell number is shown.
(TIF)

Movie S1 **Time-lapse recording of cell division progression in partial embryos.** Development of a whole embryo is shown on the left. At the 64-cell stage, B7.4 and B7.8 muscle precursor cells were isolated and recorded up to the terminal cell division. When cell division occurred, “D” appears in the top-left corner. B7.4 divided three times, while B7.8 divided twice after the 64-cell stage. Note that in both lineages, the timing of the final cell divisions was temporally close. The movie comprises one recording taken every 3 min for a total of 11 hr, covering the period from the 64-cell stage to the tailbud stage.
(AVI)
