# Supplementary figures and images for: Regulation of the Number of Cell Division Rounds by Tissue-Specific Transcription Factors and Cdk Inhibitor during Ascidian Embryogenesis
Source: PLoS One. 2014 Mar 7;9(3):e90188. doi: 10.1371/journal.pone.0090188 (PMC3946487; doi:10.1371/journal.pone.0090188)

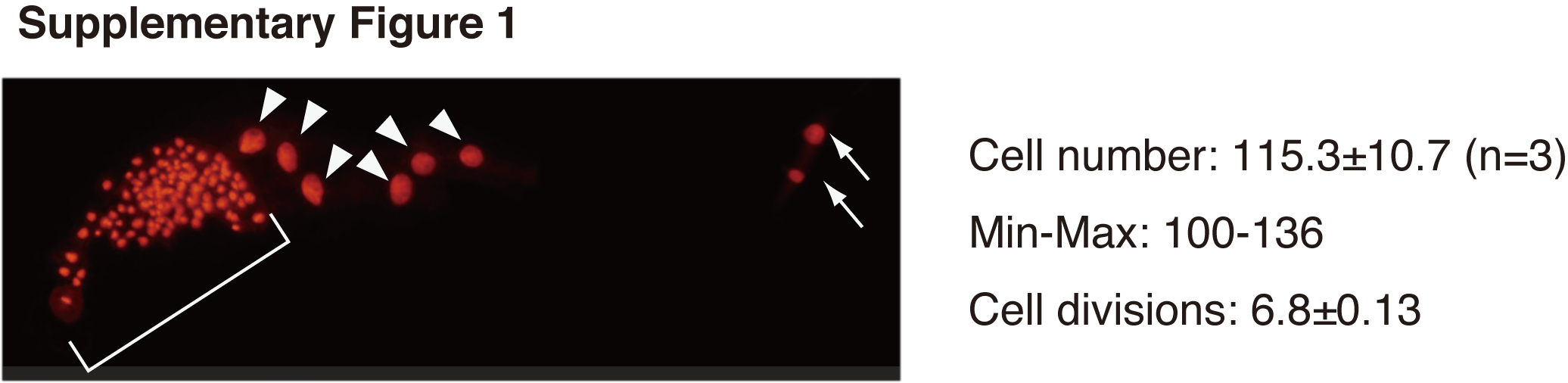

Supplement: Figure S1 — Number of descendant cells of the B7.7 mesenchyme precursor. B5.2 blastomere of the 16-cell embryo was labeled with H2B:mCherry. It gives rise to B7.7 mesenchyme (square bracket), B7.5 and B7.8 muscle cells (6 arrowheads), and B7.6 primordial germ cells (two arrows). The larva was squashed to count the number of nuclei. Mesenchyme cell number is shown. (TIF) [file pone.0090188.s001.tif]
